# Climate and *Human coronaviruses 229E* and *Human coronaviruses* *OC43* Infections: Respiratory Viral Infections Prevalence in Hospitalized Children in Cheonan, Korea

**DOI:** 10.4014/jmb.2004.04052

**Published:** 2020-08-12

**Authors:** Jang Mook Kim, Jae Sik Jeon, Jae Kyung Kim

**Affiliations:** 1 Department of Health Administration, College of Health Sciences, Dankook University, Cheonan 31116, Republic of Korea; 2 Department of Biomedical Laboratory Science, College of Health Sciences, Dankook University, Cheonan 31116, Republic of Korea

**Keywords:** Climate, HCoV-229E, HCoV-OC43, seasonal respiratory viruses, pediatric illness

## Abstract

The study of climate and respiratory viral infections using big data may enable the recognition and interpretation of relationships between disease occurrence and climatic variables. In this study, realtime reverse transcription quantitative PCR (qPCR) methods were used to identify Human respiratory coronaviruses (HCoV). infections in patients below 10 years of age with respiratory infections who visited Dankook University Hospital in Cheonan, South Korea, from January 1, 2012, to December 31, 2018. Out of the 9010 patients who underwent respiratory virus real-time reverse transcription qPCR test, 364 tested positive for HCoV infections. Among these 364 patients, 72.8% (*n* = 265) were below 10 years of age. Data regarding the frequency of infections was used to uncover the seasonal pattern of the two viral strains, which was then compared with local meteorological data for the same time period. HCoV-229E and HCoV-OC43 showed high infection rates in patients below 10 years of age. There was a negative relationship between HCoV-229E and HCoV-OC43 infections with air temperature and wind-chill temperatures. Both HCoV-229E and HCoV-OC43 rates of infection were positively related to atmospheric pressure, while HCoV-229E was also positively associated with particulate matter concentrations. Our results suggest that climatic variables affect the rate in which children below 10 years of age are infected with HCoV. These findings may help to predict when prevention strategies may be most effective.

## Introduction

Human respiratory coronaviruses (HCoV) are a broad and important group of RNA viruses that include the pathogens responsible for respiratory infections such as severe acute respiratory syndrome (SARS) and Middle East respiratory syndrome (MERS). HCoV possess unique molecular mechanisms for transcription and recombination, which lead to various etiologies and continually emerging pathogens [1]. In humans, HCoV infections primarily involve the upper respiratory and gastrointestinal tracts, and they vary from a mild, self-limiting disease, such as the common cold, to more severe manifestations, such as bronchitis and pneumonia with renal involvement [2].

There are currently no commercial vaccines against most respiratory viruses, except for influenza A and B [3], and the prevention and effective control of viral infections remain difficult. Therefore, investigations into the viral etiology of respiratory infections that occur at various ages and times play an important role in the successful implementation of prevention, control, and treatment strategies [4].

Three theories have been put forward to explain viral seasonality [5]: 1) the effect of climatic conditions on host resistance to infection (*e.g.*, low vitamin D levels following lack of sun exposure can affect the ability to fight infection) [6, 7]; 2) the effect of meteorological factors (*e.g.*, temperature and humidity) on viral survival and infection rates [8]; and 3) the effect of behavioral changes on transmission (*e.g.*, spending more time indoors and in close proximity to others or the aggregation of susceptible children at schools during the colder months) [9].

## Materials and Methods

### Sample Collection

This was a retrospective study that used respiratory virus real-time reverse transcription quantitative PCR (qPCR) results that were collected from January 2012 to December 2018, from 9010 patients who visited Dankook University Hospital in Cheonan, Korea. Collected respiratory specimens (nasopharyngeal aspirates, nasal swabs, and throat swabs) were tested immediately, otherwise they were refrigerated at 4°C and tested within 24 h. qPCR was tested by Department of Laboratory Medicine at Dankook University Hospital.

### Clinical Variables

Data on age and sex of study participants were retrieved from patient records. We were unable to collect information on the incubation period of the viruses because of the retrospective nature of the study and because the time of hospital visit after the onset of the disease was different for each patient.

### Viral Targets

The viruses under investigation were the HCoV-229E in the family Coronaviridae, genus *Alphacoronavirus*, species *Human coronavirus* 229E, and HCoV-OC43 in the family Coronaviridae, genus *Betacoronavirus*, species *Human coronavirus OC43*. The nomenclature and taxonomy of these viruses were selected according to the criteria of the International Committee on Taxonomy of Viruses).

### Ethical Considerations 

This study was approved by the Institutional Review Board Committee of Dankook University (No. 2019-12-007). This study was conducted in accordance with the Declaration of Helsinki.

### Real-Time Reverse Transcription qPCR Analysis

Viral RNA was extracted from the collected specimens using the QIAamp MinElute Virus Spin Kit (Qiagen GmbH, Germany). The extracted nucleic acids were amplified and probed for respiratory viruses with the AdvanSure RV real-time reverse transcription qPCR kit (LG Life Science Ltd., Korea) according to the manufacturer’s instructions.

### Climatic Variables 

Cheonan, Chungcheongnam-do, Korea, has a typical temperate climate of 11.8°C at 36.47° N, 127.13° E, and covers an area of 636.3 km^2^. Weather data for the Cheonan region from January 1, 2012, to December 31, 2018, was acquired from the National Institute of Environmental Research. Daily automated surface observing system (ASOS) data and particulate matter concentration data were collected.

Climate data, including dates and times for air temperature (°C), refrigeration temperature (°C), humidity (%), precipitation (mm), atmospheric pressure (hPa), and particulate matter concentration (μg/m^3^), were used to analyze the climate variables. Wind-chill temperature was assessed using the formula:

13.12 + 0.6215 × T-11.37V0.16 + 0.3965V0.16 × T

where T is the air temperature and V the wind speed (km/h) as measured 10 meters above the ground.

### Statistical Analysis 

SAS version 9.4 (SAS Institute Inc., USA) was used to perform a descriptive statistical analysis, frequency analysis, t-test, and binomial logistic regression analysis to investigate the relationship between meteorological and particulate matter concentrations with HCoV-229E and HCoV-OC43. For all analyses, statistical significance was set at *p* < 0.05.

## Results

During the study period, out of the 9,010 tested patients, 364 patients tested positive for HCoV infection, 72.8%(*n* = 265) of whom were aged 1–9 years. The average age of patients with an HCoV-229E infection and with an HCoV-OC43 infection was 2.6 years (median age 1.5 years, range 0.0–9.9 years) and 1.7 years (median age 1.1 years, range 0.0–9.6 years), respectively. There was a higher detection rate of HCoV in the 1–9 age group (53.9%, *n* = 143/265) than the <1 age group (46.1%, *n* = 122/265). Three patients were simultaneously infected with HCoV-229E and HCoV-OC43. In addition, we observed that the detection rate of other respiratory viruses was 21.3% for rhinovirus (*n* = 1920), 12.8% for respiratory syncytial virus (*n* = 1150), 9.9% for the influenza virus (*n* = 894), and 6.6% for the parainfluenza virus (*n* = 598).

**Fig. 1:**
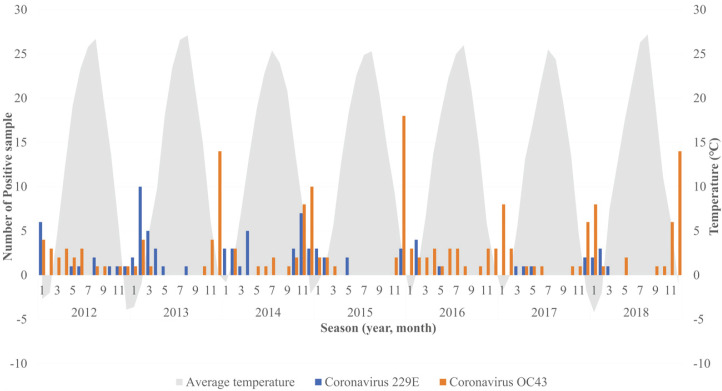
Detection-rate frequencies aggregated by month and year for *Human coronavirus 229E* and *Human coronavirus OC43* in children below 10 years of age in Cheonan, Korea.

The number of patients infected with either HCoV-229E or HCoV-OC43 sharply increased during the low-temperature winter months of October through February (Fig. 1).

A t-test analysis found that air and wind-chill temperatures, atmospheric pressure, and particulate matter concentration means were significantly different according to the days when HCoV-229E infections were detected (Table 1). The average air and wind-chill temperatures were lower on days when HCoV-229E infections were detected, and the mean differences in these two climatic variables were similar. Both atmospheric pressure and particulate matter concentration means were also significantly higher on days when viral infections were detected. No significant differences in mean precipitation or relative humidity according to infection detection rates were found.

A significant difference was noted in the mean air and wind-chill temperature according to the days when HCoV-OC43 infections were detected. Air and wind-chill temperature averages and mean differences were similar for both viruses. In contrast to HCoV-229E, the difference in means was smallest for relative humidity and largest for wind-chill temperature (mean difference 0.51, *p* = 0.606; −7.79, *p* < 0.0001, respectively) according to HCoV-OC43 viral detection days.

In the binary logistic regression analysis, air and wind-chill temperatures, air pressure, and particulate matter concentrations were significantly associated with HCoV-229E infections. Air and wind-chill temperatures showed a negative effect on HCoV-229E infections, while atmospheric pressure and particulate matter concentrations demonstrated a positive effect. That is, higher numbers of HCoV-229E infections occurred at lower air and wind-chill temperatures and higher atmospheric pressure and particulate matter concentrations. For HCoV-OC43, the infection rate was high at low air and wind-chill temperatures and high atmospheric pressure (Table 2).

## Discussion

During the study period, HCoV-229E and HCoV-OC43 infections showed an increased infection rate during the winter months. In temperate regions, the seasonality of some viruses is quite pronounced, and a number of studies have been conducted to determine the cause, but the relationship between climate and viral infections remains unclear.

In one study that used a guinea pig model, it was shown that the transmission of the influenza virus was more effective in cold and dry conditions [10]. In temperate zones, a related CoV, MERS-CoV, is known to be affected by air humidity variables, and the incidence of MERS is known to increase between April and August. Low wind speed and low relative humidity are contributors to increased MERS-CoV cases [11]. In contrast, there have been reports that increased inactivation and decreased survival of the SARS virus are associated with higher surface temperatures in the environment [12]. Recently, Mao et al. reported that temperatures could significantly affect COVID-19 transmission and there might be an optimal temperature for the viral transmission, which may partly explain why the pandemic of SARS-CoV-2 first broke out in Wuhan [23]. In this study, air and wind-chill temperatures and atmospheric pressure were significantly associated with both HCoV-229E and HCoV-OC43 infections. In particular, air and wind-chill temperatures appeared to affect the infection rates of both viruses negatively. This may be due to temperature having an important effect on the activity of enveloped viruses, especially influenza and coronaviruses.

The relationship between particulate matter 10 µg/m^3^ in diameter or less (PM_10_) and respiratory viruses in the atmosphere may be difficult to detect due to technical limitations. However, there have been studies on the association between PM_10_ and respiratory viral infections [5,17,20-23]. In particular, increases in PM_10_ may pose problems for those with pre-existing conditions. For instance, it increases hospitalizations [13], worsens respiratory symptoms, and lowers lung functioning in asthma patients [14]. For patients with chronic obstructive pulmonary disease, it increases hospitalizations [15] and infections with viral influenza and pneumonia [16]. In diabetic patients, increased mortality from hyperinfluenza virus infections [17], cardiovascular and lung diseases, cancer, and increased cardiovascular hospitalizations [18] have occurred with increases in PM_10_; in children, there are increased outpatient visits [19]. In this study, we observed increases in HCoV-229E infection with increases in particulate matter concentrations. This is consistent with reports in the literature showing increases in the rates of hospital visits by respiratory patients when particulate matter concentrations are increased [19, 21, 22]. This may be due to increasing PM_10_ concentrations that cause an increase in oxidative stress and epithelial cell permeability, along with inactivation of macrophages and decreases in T-cell functioning, which would allow for increases in viral infectivity [17].

There were several limitations to this study. First, a 7-year investigation in a single city is a relatively short amount of time and is much more localized than other long-term studies and/or investigations that have been undertaken using national surveillance programs. Hence, a bias in viral etiology may exist.

Second, this study may have included those who did not live in the catchment area. As the data was anonymized, it was not possible to determine whether the individuals were visitors or residents of the area. However, all samples tested over the study time were analyzed, and we believe that the sample was large enough to overcome potential sampling error due to the possible inclusion of non-residents.

Despite these limitations, this study may be used as a basis for further studies as our data links medical and meteorological data for specific respiratory viral infections occurring in the same region over a 7-year time period. In addition, this is the first study to include the climatic variables particulate matter concentrations and wind-chill temperatures and to confirm that HCoV-229E infections increase concomitantly with particulate matter concentrations [24, 25].

Our results may also help to better understand the distribution of viruses over different seasons and patient age ranges, which could assist in the development of effective prevention strategies for respiratory viral infections, support systematic treatment of respiratory sensitization directly related to climate, and inform potential treatment approaches such that they directly take into account climatic variables.

## Figures and Tables

**Table 1:** The mean difference in climatic variables according to days when *Human coronavirus 229E* and *Human coronavirus OC43* infections were detected.

Virus	Climatic variables	Mean climatic values on days the virus was		Difference in means	95% CI		*p*-value^*^
		Lower	Upper		Detected	Not detected	
Coronavirus 229E	Air temperature (°C)	5.09	11.70	-6.61	-8.74	-4.47	**<0.0001**
	Wind-chill temperature (°C)	4.07	11.13	-7.06	-9.32	-4.79	**<0.0001**
	Relative humidity (%)	65.06	67.47	-2.41	-5.17	0.35	0.087
	Precipitation (mm)	3.38	2.71	0.68	-1.62	2.97	0.564
	Atmospheric pressure (hPa)	1015.2	1007.6	7.7	4.59	10.74	**<0.0001**
	Particulate matter (μg/m^3^)	63.15	50.52	12.63	6.58	18.67	**<0.0001**
Coronavirus OC43	Air temperature (°C)	4.36	11.82	-7.46	-8.95	-5.98	**<0.0001**
	Wind-chill temperature (°C)	3.47	11.26	-7.79	-9.36	-6.22	**<0.0001**
	Relative humidity (%)	67.93	67.42	0.51	-1.42	2.44	0.606
	Precipitation (mm)	1.67	2.75	-1.08	-2.68	0.52	0.186
	Atmospheric pressure (hPa)	1008.4	1007.7	0.8	-1.40	2.91	0.492
	Particulate matter (μg/m^3^)	53.57	50.62	2.95	-1.37	7.27	0.181

^*^Significant *p*-values in bold. CI, Confidence Intervals

**Table 2:** Correlations between climatic factors with *Human coronavirus 229E* and *Human coronavirus OC43* infections.

Virus	Climatic variables	*p*-value^*^	Odds ratio	95% CI	
				Lower	Upper
Coronavirus 229E	Air temperature (°C)	**<0.0001**	0.404	0.280	0.585
	Wind-chill temperature (°C)	**<0.0001**	0.491	0.339	0.710
	Relative humidity (%)	0.429	0.862	0.596	1.246
	Precipitation (mm)	0.192	0.719	0.439	1.180
	Atmospheric pressure (hPa)	**<0.0001**	2.821	1.950	4.082
	Particulate matter (μg/m^3^)	**<0.0001**	2.263	1.564	3.274
Coronavirus OC43	**<0.0001**	<0.0001	0.542	0.419	0.702
	Wind-chill temperature (°C)	**<0.0001**	0.594	0.459	0.769
	Relative humidity (%)	0.538	1.084	0.838	1.403
	Precipitation (mm)	0.699	0.938	0.680	1.295
	Atmospheric pressure (hPa)	**<0.0001**	2.105	1.626	2.725
	Particulate matter (μg/m^3^)	0.124	1.230	0.945	1.600

^*^Significant *p*-values in bold. CI, Confidence Intervals
